# Effectiveness of a computerized drug-monitoring program to detect and prevent adverse drug events and medication non-adherence in outpatient ambulatory care: study protocol of a randomized controlled trial

**DOI:** 10.1186/1745-6215-16-2

**Published:** 2015-01-08

**Authors:** Alan J Forster, Tobias E Erlanger, Alison Jennings, Claudine Auger, David Buckeridge, Carl van Walraven, Robyn Tamblyn

**Affiliations:** Clinical Epidemiology Program, Ottawa Hospital Research Institute, 1053 Carling Avenue, Ottawa, ON K1Y 4E9 Canada; The Ottawa Hospital, 1053 Carling Avenue, Ottawa, ON K1Y 4E9 Canada; Department of Epidemiology and Community Medicine and Department of Medicine University of Ottawa, 451 Smyth Road, Ottawa, ON K1H 8M5 Canada; Institute for Clinical Evaluative Sciences – Ottawa (ICES@uOttawa), ASB-1, 1053 Carling Avenue, Ottawa, ON K1Y 4E9 Canada; Department of Epidemiology, Biostatistics and Occupational Health, McGill University, 1020 Pine Avenue West, Montréal, QC H3A 1A2 Canada; Clinical and Health Informatics Research Group, McGill University, 1140 Pine Avenue West, Montréal, QC H3A 1A3 Canada; School of Rehabilitation, Université de Montréal, C.P. 6128, succursale Centre-ville, Montréal, QC H3C 3J7 Canada; Center for Interdisciplinary Research in Rehabilitation of Greater Montreal (CRIR), site Centre de Réadaptation Lucie-Bruneau, 2275 Avenue Laurier Est, Montréal, QC H2H 2N8 Canada; Department of Medicine, Royal Victoria Hospital, McGill University, 687 Pine Avenue West, Room A3.09, Montréal, QC H3A 1A1 Canada

**Keywords:** Patient safety, Pharmacoepidemiology, Adverse drug events, Drug adherence and persistence, Health information technology, Health services research

## Abstract

**Background:**

Medications are an effective intervention for managing and preventing health problems but their benefit can be undermined by non-adherence or adverse drug events (ADEs). Since these issues may be interconnected, efforts to improve non-adherence should also include reduction of ADEs. We have developed the ISTOP-ADE system (Information Systems-enabled Outreach for Preventing Adverse Drug Events), which enables timely monitoring and managing of ADEs. The objectives of this study are to determine whether the ISTOP-ADE system, compared to routine care, will reduce: a) the probability of discontinuing the use of prognosis-altering medications; b) the probability of a patient experiencing a severe ADE; c) the proportion of patients experiencing ADEs, preventable ADEs and ameliorable ADEs; and d) health services utilization.

**Methods/design:**

We will randomly assign 2,200 adult ambulatory patients in the province of Québec who have been prescribed an incident medication for the management or prevention of a chronic health condition, to routine care or the ISTOP-ADE system. The ISTOP-ADE system consists of an interactive voice response system (IVRS) paired with pharmacist support. The IVRS will call patients at 3 and 17 days post-prescription to determine if they are experiencing any problems and connect them with a pharmacist when required or desired by the patient. We will evaluate medication persistence at 180 days and health-care utilization using provincial administrative data. Two blinded physicians will ascertain ADE status through a case review.

**Discussion:**

We expect the ISTOP-ADE intervention to be feasible and to improve the quality of patient care through improved medication adherence, reduced ADE duration and reduced number of ADEs resulting in an emergency department or inpatient encounter. This in turn could lower health-care utilization, saving costs and lowering the burden on emergency departments and family practices. The success of ISTOP-ADE would present opportunities to implement this intervention through health systems, health insurance agents and commercial pharmacies.

**Trial registration:**

ClinicalTrials.gov Identifier: NCT02059044. Date registered: 10 January 2014.

## Background

Medications are an effective intervention for managing and preventing many acute and chronic health-care problems. Their benefit is commonly reduced by non-adherence, in which patients either do not take or stop taking the medication as prescribed, or by adverse drug events (ADEs), in which patients experience side effects of the medications. These issues are interrelated. Some patients do not take their medication due to their fear of an impending ADE, while others stop taking the medication because of an ADE. Therefore, efforts to increase adherence should include efforts to target ADE reduction specifically.

To address these concerns, we have developed the Information Systems-enabled Outreach for Preventing ADEs (ISTOP-ADE) system (protocol version 1, 1 March 2012). The ISTOP-ADE system consists of a set of business processes supported by a technology infrastructure. The information technology is designed to connect providers to patients and includes an electronic health record system, a clinical data repository and an interactive voice response system (IVRS). This system enables timely monitoring of patients for ADEs and couples it with an effective arm to manage ADEs if, and when, they occur.

We recently established the feasibility and acceptability of the ISTOP-ADE system in a prospective cohort study. The pilot study included 628 patients who were prescribed an incident prescription. The ISTOP-ADE system successfully contacted 90% of patients. Important measures of the system’s effectiveness included the identification of 45% of ADEs, 50% of all potential ADEs and 30% of primary non-adherence episodes. In terms of potential benefits, it positively influenced the management of 42% of all medication-related problems. In terms of acceptability, almost all patients found it easy to use and understand and reported they would use the system again if it was provided to them. Physicians and pharmacists also supported the system [1, 2]. While these results are favorable, a controlled clinical trial in which patients are randomly assigned to the ISTOP-ADE system or routine care is required to determine the system’s effectiveness. We therefore designed this study.

## Methods/design

This study has been approved by the McGill Institutional Review Board (A01-B02-02A) and the Ottawa Health Science Network Research Ethics Board (20130285-01H).

### Setting

This research will occur during ambulatory care in the province of Québec, Canada. We will study prescriptions in patients receiving primary care from a selected group of physicians using the Medical Office of the 21st Century (MOXXI) electronic health record system. MOXXI was developed by the McGill University’s Clinical and Health Informatics Research Group. It is currently used by over 100 primary care physicians in Montréal and Québec City. The MOXXI system is linked to the provincial insurance company, Régie de l’assurance maladie du Québec (RAMQ), resulting in an electronic medical record that displays a drug profile of prescribed as well as dispensed drugs, emergency department visits and hospitalizations in the past 12 months, a list of active and past health problems, and an electronic prescribing tool. Data stored by MOXXI have been used by the McGill research team for medical research in the past [3, 4].

### Primary objective

The primary objective is to determine whether the ISTOP-ADE system, compared to routine care, will reduce the probability of discontinuing the use of prognosis-altering medications in patients prescribed an incident medication to manage or prevent a chronic health condition.

### Secondary objectives

- Determine whether the ISTOP-ADE system, compared to routine care, will reduce the probability of a patient experiencing a severe ADE (defined as an ADE lasting at least 7 days or causing an emergency department visit).
- Determine whether the ISTOP-ADE system, compared to routine care, will reduce the proportion of patients experiencing ADEs, preventable ADEs and ameliorable ADEs.
- Determine whether the ISTOP-ADE system, compared to routine care, will reduce health services utilization (comprising physician visits, emergency department encounters and hospitalizations) at 12 months following an incident prescription.
- Measure the costs associated with the ISTOP-ADE system.

### Participants

This study will include French- and English-speaking adult patients (age >18) who receive a high-risk incident prescription, use RAMQ insurance to pay for medications and are followed by a physician who has consented to be in the MOXXI research network. We will exclude patients if their physicians do not deem them appropriate for study inclusion.

We define high-risk prescriptions as medications commonly used to prevent progression or complications related to the following conditions: congestive heart failure, hypertension, dyslipidaemia, type 2 diabetes mellitus, coronary artery disease, cerebrovascular disease, atrial fibrillation, chronic renal failure, chronic obstructive lung disease, depression, dementia, autoimmune conditions, seizures, and chronic pain. The selected medications (Table 1) have an important impact on outcomes or they have been shown to have a high frequency of ADEs [5, 6]. We will consider prescriptions as ‘incident’ if the patient had not received a prior prescription for that medication in the 12 months preceding the index event.

**Table 1:** **Included diseases and drug classes for the ISTOP-ADE study**

Disease entity	Drug classes included
Congestive heart failure	Angiotensin-converting enzyme inhibitors, angiotensin II receptor antagonists, hydralazine, mineralocorticoid (aldosterone) receptor antagonists, nitrate, diuretic, cardiac glycoside, β-adrenergic blocking agents
Diabetes mellitus	Sulfonylurea/insulin secretagogue, biguanide, A-glucosidase inhibitor thiazolidinedione, incretin therapy (dipeptidyl peptidase-4 inhibitor), insulin
Hypertension	β-adrenergic blocking agents, diuretic, calcium-channel blocking agents, angiotensin-converting enzyme inhibitors, angiotensin II receptor antagonists, nitrate, central α-agonists
Coronary artery disease	β-adrenergic blocking agents, statin, calcium-channel blocking agents, angiotensin-converting enzyme inhibitors, angiotensin II receptor antagonists, nitrate, acetylsalicylic acid, clopidogrel
Dyslipidemia	Statin, cholestyramine resin, fibric acid derivatives, niacin derivative, cholesterol absorption inhibitor
Cerebrovascular disease	Acetylsalicylic acid, clopidogrel, statin, dypiramidole
Atrial fibrillation	β-adrenergic blocking agents, cardiac glycoside, class III anti-arrhythmics, class Ic anti-arrhythmics, calcium-channel blocking agents, vitamin K antagonist, thrombin inhibitor, Acetylsalicylic acid
Chronic kidney disease	Angiotensin-converting enzyme inhibitors, angiotensin II receptor antagonists, thiazide diuretic, β-adrenergic blocking agents, calcium-channel blocking agents
Chronic obstructive pulmonary disease	Short-acting bronchodilators, long-acting bronchodilators, phosphodiesterase type-4 inhibitors, combination inhaled corticosteroids and long-acting bronchodilators, theophylline
Depression	Serotonin-specific reuptake inhibitors, serotonin-norepinephrine reuptake inhibitors, monoamine oxidase inhibitors, tricyclic antidepressants, quetiapine, bupropion, trazodone, mirtazapine
Dementia	Parasympathomimetic (cholinergic)
Autoimmune conditions	Corticosteroids
Seizures, chronic pain	Anticonvulsants

Participants will be enrolled in the ISTOP-ADE study if they have an incident prescription for a high-risk medication. We define high-risk prescriptions as medications commonly used to prevent progression or complications related to key conditions (disease entities). The selected drug classes have an important impact on outcomes or they have been shown to have a high frequency of ADEs.

### Eligibility assessment and consent

When a physician uses MOXXI to prescribe a new medication for a patient, a pop-up window will appear on their computer screen to indicate the patient’s eligibility. This pop-up window will prompt the doctor to inform the patient of the study, confirm their eligibility and seek consent. We will obtain informed consent from each participant. If the patient consents, the physician will confirm the patient’s phone number, preferred language and preferred time of day to be called. If the physician deems the patient ineligible or the patient does not consent, then the reason will be captured in MOXXI using a standard classification.

### Randomization

The unit of analysis for the study is the individual patient, each of whom we will randomly assign to the intervention or control arms using a computer algorithm. We will perform stratified randomization at the physician practice level with block sizes of four. We will only randomize patients once a physician confirms the patient’s eligibility for enrolment. We will update the ISTOP-ADE system with the patient’s treatment group but this information will not be available to the prescribing physician or the patient.

### Intervention

The intervention consists of an IVRS call coupled with pharmacist support. We will program the IVRS to call patients automatically on day 3 and day 17 following their prescription. If a call is not answered, the system will make as many as eight separate attempts (two per day over a maximum of 4 days) before marking the call as unsuccessful. If a call is answered, the IVRS will proceed through a calling algorithm. The following questions will be asked at each call:

1. Since receiving your medication, have you started taking it?
2. Since receiving your medication, have you had any problems?
3. Since receiving your medication, have you developed any new symptoms?
4. Would you like to speak to a pharmacist?

If the patient answers ‘No’ to the first question or ‘Yes’ to any of the three remaining questions, the IVRS will inform the patient that a pharmacist will personally contact them within 2 business days. The IVRS will send an email to a pharmacist informing them of the patient’s request. In addition, the IVRS will be capable of receiving inbound calls from patients regarding their medications at any time. This will also connect the patient to the pharmacist according to the same business rules as for the outbound IVRS calls.

When required, the pharmacist will contact the patient. Prior to contact, the pharmacist will access the electronic health record to obtain background information on the patient’s health status and medication use. During the call, the pharmacist will document the patient’s problems as well as their recommendation for resolving the problem in the electronic health record. If the pharmacist deems the patient’s problems are significant, then the pharmacist can escalate the issue to the patient’s primary care physician using the electronic health record.

### Control

The control arm consists of routine care.

### Instrumentation

#### Interview at 21 days

We will perform a standard interview of patients at 21 days to determine the development of new symptoms, medication use and health services use since their prescription. The study personnel performing the interview will not have access to knowledge regarding the study group to which the patient is allocated.

#### Administrative database interrogation

We will link the study database to the drug benefit and hospital record files contained at RAMQ using the patient’s provincial health insurance number.

### Outcome assessment

#### Primary outcome: medication persistence at 180 days

We define persistence as ‘having a continuous supply of at least one medication used to treat the index condition, with gaps no greater than 90 days, in the first 6 months of treatment’. We will use validated methods to measure medication persistence using administrative claims data on prescription refills [7–11]. Prescription refills provide a good proxy measure of medication use because refill rates are strongly correlated with targeted clinical outcomes such as blood pressure [7]. We will construct medication dispensing histories for all patients and medications during the study follow-up period using the RAMQ medication file.

#### Secondary outcome: ADEs within 21 days

We will use previously developed methods to ascertain ADE status [12–17]. Specifically, a clinician will create a case summary describing the post-prescription course for each patient experiencing a problem. Problems will be identified during the 21-day interview. The clinician will compile information derived from the post-intervention interview and a review of the patient’s MOXXI record using a standardized format. The summary will elaborate on the patient’s underlying health conditions, the medications currently being used (including the index prescription), the timing of the problem, the management of the problem, the timing of the resolution and what the patient was told about the cause of the problem.

To define ADE status, two physicians (blinded to allocation group) will review each case summary and determine whether the case represents an ADE. This determination is made using a six-point classification system with a cut-point of 3. A case is considered to be an ADE if both reviewers agree that the case was an undesirable outcome and that outcome was more likely caused by medication use than the patient’s underlying health condition. This method has been widely adopted as the standard approach for ADE classification in patient safety research [12–17]. For an ADE, the reviewers will further classify its duration, preventability, amelioration and severity using standard definitions [12–19].

#### Health services utilization

Standard methods to measure health services use using provincial claims data will be used. All physician visits, emergency department visits, hospitalizations and medication claims during the 12 months following index prescription will be identified using the RAMQ data through MOXXI.

#### Intervention costs

We will record the entire amount of time the study pharmacist spends supporting patients in the intervention group. This will allow us to calculate costs at the patient level as well as aggregated at the population level. We will also track implementation and support costs for the IVRS, including calling charges. These costs will be prorated across the study population.

#### Explanatory variables

To determine factors that influence outcomes, we will use data in the MOXXI system to derive baseline information on patients, including demographic information (age, gender, language of choice and location of residence), active and past medical diagnoses and medication use in the preceding year (number of new drug starts and adherence).

### Blinding

Due to the nature of the study, blinding of patients and health-care providers will not be possible. However, we will blind personnel performing the interviews and physicians performing the assessment of ADE status of study group allocation.

### Sample size

We have based our sample size on the following assumptions:

- A baseline probability of non-persistence of 25% (based on a prior research study evaluating persistence with anti-hypertensive therapy using the same methods we will for this study) [11]
- A minimally clinical important difference of 20%
- A 10% loss to follow-up (based on our pilot study)
- An enrollment rate of 50% (based on our pilot study)
- Other specifications: α = 0.05, β = 0.20 and two-sided tests for significance will be used

Given these assumptions, we will need to approach 4,708 patients in total (2,354 patients in each treatment arm being eligible and approached for enrollment). This will result in 2,354 patients being enrolled (1,177 patients in each arm) and 2,118 patients evaluated for the composite outcome (1,059 patients in each arm). To be more conservative, we will enroll 1,100 patients in each arm.

### Data management and analysis

Data will be exported from the MOXXI system and analyzed using SAS software (Cary, NC, USA). All data analysis will be performed in secure physical locations using hardware that is password protected. Only study personnel will have access to the data. Simple descriptive statistics to describe the patient population, call statistics and outcome measures will be used. Continuous variables will be described using median and inter-quartile ranges, while categorical variables will be described using frequency distributions. The statistical significance of any associations between group assignment and measures will be assessed using the Wilcoxon rank sum test and or the chi-square test, respectively, for continuous and categorical variables. Since the primary outcome is binary, tests to compare outcomes between the intervention and control groups will be simple chi-square statistics. An alpha of 0.05 as a threshold to determine statistical significance using a two-tailed distribution will be applied.

As a sensitivity analysis, logistic regression modeling will be used to assess possible baseline factors that are associated with the outcome and differ between the intervention and control groups. Analogous analyses to assess significance for the individual components of the composite primary outcome and the binary secondary outcome variables will also be performed (ADE occurrence, preventable ADE occurrence and ameliorable ADE occurrence).

To compare health services utilization, since the outcomes are event data, which follow a Poisson distribution, incidence rates for the outcome will be calculated. Incidence rates will be calculated using PROC GENMOD to create a *t*-test. Multivariable Poisson regression will be used to compare the rates, while adjusting for baseline factors. The model will use the treatment group as an independent covariate. If over-dispersion is noted, a negative binomial model will be used.

### Ethical considerations

The physicians and their patients enrolled in the MOXXI program are covered under an umbrella consent program, which is approved and monitored by McGill’s Institutional Review Board. In addition, patients will be given opportunities to become informed about the project and to withdraw, as appropriately defined under the Tri-Council’s guidelines.

### Timeline

The entire ISTOP-ADE study will take 5 years from start to finish. Patient enrolment will run for 3 years to ensure sample size requirements are met. Patient follow-up will continue for 1 year from enrolment. Case summaries and physician reviews, as well as data validation and quality checks will occur throughout the lifespan of the study. We have budgeted 12 months to accomplish the analysis and knowledge translation activities.

## Discussion

We have planned this randomized trial to assess the effectiveness and cost-effectiveness of an automated outreach program to monitor patients receiving a new ambulatory care prescription. It is an important study as the intervention has shown promising results in a pilot study. Some health payers and pharmacies may feel that this intervention is already warranted on face value and based on our preliminary findings. We have advised against this approach as the system has not been compared against routine care. In a situation where there are limited resources, it is not appropriate to invest in interventions of unknown effectiveness.

We chose adherence with prognosis-altering medications as our primary outcome. This outcome is important as many studies have demonstrated poor adherence – approximately 50% of patients with chronic illness do not take their medication as prescribed – and this in turn has been estimated to have major impacts on disease progression and health-care costs [20, 21]. We will assess this outcome using administrative data. This ascertainment method has been demonstrated to be accurate and will not be biased by an inability to blind patients to the intervention. Our secondary outcomes are similarly important. In particular, ADEs are common following incident prescriptions [15, 16]. While many of the symptoms related to ADEs are short in duration, a substantial proportion of patients have symptoms lasting longer than 7 days and can lead patients to discontinue their medications on their own. Collectively, these outcomes will allow us to assess adequately the value of our intervention to patients and the health-care system.

Our study will also give us an opportunity to assess further implementation issues. We will track the costs of the intervention closely, including the workload required by our pharmacist to follow up the automated calls and by the physicians, who may need to do extra work to respond to patient complaints. We will also assess the failed automated calls to assess which patients do not accept this type of intervention. Finally, we will conduct an assessment of patient factors associated with adherence. All of this information will guide decisions pertaining to whether and how this system should be implemented, and to whom it should be directed.

This is an ambitious study, which will run in over 100 primary care practices in two large urban settings. It will take 3 years to complete the recruitment and follow-up. We are randomizing patients at the patient rather than provider level because there is little risk of contamination or co-intervention. The patients are unlikely to interact with each other, and, based on our pilot study, there was very little interaction between patients and doctors within the 3 weeks of their prescription. There are some sources of bias in our design to be considered. The primary risk is the lack of blinding in patients and primary care doctors. This is a concern as knowledge of allocation could change behavior. However, based on findings from our pilot study, we predict this is unlikely. In addition, outcome assessment is done objectively or by blinded reviewers. In short, this study will have internal validity and will be generalizable.

In summary, we have previously developed an automated outreach program for patients prescribed new medications. We have tested this intervention in a pilot study. This work found the intervention to be feasible and suggested it might improve the quality of patient care through reduced ADE duration and improved medication adherence. We have designed this randomized trial to test whether the intervention is effective compared to routine care. If the ISTOP-ADE system were demonstrated to be effective, then there would be significant opportunities to implement this intervention through health systems, health insurance agents and commercial pharmacies.

## Trial status

The ISTOP-ADE study is anticipated to start early 2015.

## Abbreviations

ADE: adverse drug event
ISTOP-ADE: Information Systems-enabled Outreach for Preventing ADEs
IVRS: interactive voice response system
MOXXI: Medical Office of the 21st Century
RAMQ: Régie de l’assurance maladie du Québec.
